# Efficacy and Safety of *Zataria multiflora* Boiss Essential Oil against Acute Toxoplasmosis in Mice

**Published:** 2020

**Authors:** Hossein MAHMOUDVAND, Amir TAVAKOLI KARESHK, Mohammad NABI MORADI, Lianet MONZOTE FIDALGO, Seyed Reza MIRBADIE, Massumeh NIAZI, Mehrdad KHATAMI

**Affiliations:** 1. Razi Herbal Medicines Research Center, Lorestan University of Medical Sciences, Khorramabad, Iran; 2. Department of Laboratory Sciences, School of Allied Medicine, Lorestan University of Medical Sciences, Khorramabad, Iran; 3. Infectious Diseases Research Center, Birjand University of Medical Sciences, Birjand, Iran; 4. Department of Parasitology, Institute of Tropical Medicine “Pedro Kourí”, La Lisa, Havana, Cuba; 5. School of Medicine, Shahroud University of Medical Sciences, Shahroud, Iran; 6. Student Research Committee, Lorestan University of Medical Sciences, Khorramabad, Iran; 7. Bam University of Medical Sciences, Bam, Iran

**Keywords:** Prophylactic, Essential oil, *Toxoplasma gondii*, Treatment, Herbal medicines, Toxicity

## Abstract

**Background:**

Broad spectrums of pharmacological properties, including antimicrobial activity have been attributed to *Zataria multiflora* Boiss (Laminaceae). The in vivo efficacy and safety of *Z. multiflora* essential oil (ZM-EO) were evaluated against acute toxoplasmosis caused by *Toxoplasma gondii* (Sarcocystidae) in mice.

**Methods:**

*Z. multiflora* (aerial parts) was obtained from the rural districts of Kerman city (Kerman Province) Southwestern Iran, in May of 2016. Male NMRI mice were orally treated with normal saline (control group) and ZM-EO at the doses of 0.2 and 0.4 mL/kg once a day for 14 d (8 mice in each group)*.* On the 15^th^ day, the mice were infected with 104 tachyzoites of *T. gondii* RH strain by intraperitoneal route. The mortality rate and parasite load were determined in the infected mice. Additionally, 24 mice were applied to examine the sub-acute toxicity of ZM-EO at the above doses after treatment during 14 d.

**Results:**

GC/MS analysis displayed that the key constituents were thymol (45.4%), carvacrol (23%) and *p*-cymene (10.6%), respectively. Overall, 100% mortality was observed on the 8^th^ and 9^th^ days in treated mice with the concentrations of 0.2 and 0.4 mL/kg, respectively. The mean number of tachyzoites in the mice treated with 0.2 and 0.4 mL/kg of ZM-EO were 189×10^4^ and 76×10^4^ cell/mL, respectively, meaningfully (*P*<0.05) reduced compared with the control mice. Results also demonstrated that ZM-EO had no important toxicity on mice.

**Conclusion:**

The results demonstrated the efficacy of ZM-EO against acute toxoplasmosis. Nevertheless, supplementary surveys are mandatory to examine its precise effects, mainly immunomodulatory effect on toxoplasmosis.

## Introduction

*Toxoplasma gondii* (Sarcocystidae) is an opportunistic intracellular parasite found worldwide; it can involve broad-spectrum animals and a high percentage of the human population (1). The main ways of human infection with *T. gondii* include ingestion of poorly cooked or raw meat containing tissue cysts, intake of food or water contaminated with oocysts and transplacental transmission from mother to fetuses (2). Toxoplasmosis represents a few symptoms in immunocompetent individuals; however, serious complications such as injury to the brain, eyes, and other vital organs could be observed in the fetus of pregnant women and in people with a weakened or compromised immune system, including transplant recipients, patients with acquired immunodeficiency syndrome (AIDS), patients with T lymphocyte deficiency which may have lymphomas or acute lymphocytic leukemia (3–6).

The combination of pyrimethamine and sulfadiazine is currently the recommended treatment against toxoplasmosis (7). However, the use of these drugs is a challenge because of severe complications such as osteoporosis, and teratogenic effects, especially in immunocompromised patients (8). Thus, it would be beneficial to develop new treatments that have high efficacy and lack the side effects of the above-mentioned drugs for the treatment of toxoplasmosis.

Medicinal plants and their phytoconstituents have been used as a supplementary health care system for the prevention and treatment of a number of diseases such as infectious long before the discovery of the current modern drugs (9). *Zataria multiflora* Boiss. (Laminaceae) which generally cultivated in Iran (10) have a broad spectrum of pharmacological properties such as immunostimulant, antinociceptive, anti-inflammatory and antioxidant (11). Furthermore, previous investigations had demonstrated antibacterial, antiviral, antifungal, and antiparasitic effects of various parts of this plant (11, 12). The main constituents of *Z. multiflora* essential oil (EO-ZM) are monoterpenoid derivatives (11, 13). However, a number of factors, e.g., the geographical source of plant and harvesting time might be affecting the composition and biological effects of essential oils (14, 15).

The present investigation was designed to evaluate the efficacy and safety of EO-ZM on the mice infected with acute toxoplasmosis.

## Materials and Methods

### Plant materials

*Z. multiflora* (aerial parts) was obtained from the rural districts of Kerman city (Kerman Province) Southwestern Iran, in May of 2016.

Identification of the plant was done by a botanist (Prof. Fariba Sharififar) at the Kerman University of Medical Sciences, Kerman, Iran. A voucher sample was committed at the herbarium of the Kerman University of Medical Sciences (KF.1375).

### Extraction of essential oil

Hydrodistillation of the plant air-dried aerial parts was done using an all-glass Clevenger type apparatus for 3 h, the obtained essential oil was kept at 4 °C in up to use (16).

### Gas chromatography/mass spectrometry (GC/MS) analysis of essential oil

GC analysis was conducted using Hewlett-Packard 6890 (Hewlett-Packard, Palo Alto, CA) with an HP-5MS column (30 m × 0.25 mm, film thickness 0.25 mm) with the specifications and features described earlier (17). The essential oil compositions were recognized by comparing their relative retention time and mass spectra with the standards, or those described in the previous information (18).

### Parasite preparation

*Toxoplasma gondii* virulent RH strain was prepared by the Department of Parasitology and Mycology, the Kerman University of Medical Sciences, Kerman, Iran. The *Toxoplasma* tachyzoites were harvested by serial intraperitoneal passages in mice. Parasites (1 × 10 ^4^ /mL) were inoculated to the mice, and the tachyzoites were obtained after 72 h. Then, the tachyzoites were cultured and recovered with PBS and used in the tests. The tachyzoites of *T. gondii* RH strain (1 × 10 ^4^ /mL) were inoculated intraperitoneally (100 μL) to the mice in order to establish an animal model of acute toxoplasmosis.

### Experimental animals

Forty-eight male NMRI mice (40–45 d old) weighting 20–25 gr were acquired from the Pasteur Institute, Tehran, Iran. Mice were kept in a colony room with conditions of 12/12 h cycle at room temperature. The survey was agreed by the Ethical Committee of the Lorestan University of Medical Sciences (LUMS.REC.2016.148).

### Experimental design

The animals were distributed into 6 groups (8 mice /group) including:

Group 1: non-infected non-treated control group.
Group 2: infected saline-treated control group.
Group 3: non-infected treated control group with the dose of 0.2 ml/kg ZM-EO (for 2 wk).
Group 4: non-infected treated control group with the dose of 0.4 ml/kg ZM-EO (for 2 wk).
Group 5: infected treated group with the dose of 0.2 ml/kg ZM-EO (for 2 wk).
Group 6: infected treated group with the dose of 0.4 ml/kg ZM-EO (for 2 wk).

At the 15th day, the mice (2, 5, and 6 groups) were infected with 1 × 10 ^4^ /mL tachyzoite of *T. gondii* RH strain IP; the infection was observed after three days. Then, the following parameters were carried out to evaluate the efficacy of oral administration of ZM-EO against acute toxoplasmosis.

### Parasitology study

The subsequent factors were conducted for the tested mice (5 and 6 groups) in order to compare with their related control (group 2).

### Mortality rate (MR)

The subsequent formula was applied to calculate the MR:

MR=(number of dead mice/total number of tested mice×100)(19).

### Parasite load

The average number of tachyzoites was counted in the aspirated peritoneal liquid of the infected mice using a slide under a light microscope (Olympus CX31, Japan) (19).

### Clinical chemistry and hematological study

The toxicity effects of EO-ZM were evaluated by the following procedure; the mice in 3 and 4 groups in comparison with the mice in control group (Group 1) were fasted overnight. The animals were anesthetized by ketamine (100 mg/kg)-xylazine (10 mg/kg) and their thoracic cavity was opened. Blood samples (1.5–2 mL) were collected from the mice heart into cylinders containing ethylenediaminetetraacetic acid (EDTA) anticoagulant and into tubes having no anticoagulant for hematological clinical and chemistry parameters, respectively. Some parameters of hematology such as hemoglobin, hematocrit, white blood cell (WBC), red blood cell (RBC) and platelet counts, were recorded by a Hematological Analyzer, SYSMEX XT-1800i (SYSMEX Co. Japan). Additionally, Aspartate aminotransferase (AST), alanine aminotransferase (ALT), alkaline phosphatase (ALP), creatinine (Cr), blood urea nitrogen (BUN), and bilirubin (direct and total) were assessed by COBAS INTEGRA-400 Analyzer (Roche Diagnostics, Germany) on 500 μL of serum samples (17).

### Statistical analysis

Analysis of obtained data was made by SPSS statistical software 17.0 (Chicago, IL, USA). One-way ANOVA with Tukey’s post hoc test was also applied to evaluate the variations between the tested groups. Moreover, *P*<0.05 was measured as statistically significant.

## Results

### GC/MS analysis of essential oil from Z. multiflora

*Z. multiflora* yielded 3.1% v/w yellow-colored essential oil. The findings of GC/MS analysis EO-ZM are shown in Table 1. Twenty-three constituents were recognized, indicating 98.8% of the EO-ZM. The key constituents were thymol (45.4%), carvacrol (25.96%) and *p*-cymene (10.6%), respectively.

**Table 1:** Composition of essential oil composition from *Z. multiflora* identified by GC-MS

***Components***	***% Composition***	***RI^a^***
2-Nonanol	0.19	1101
Borneole	0.24	1176
trans-Sabinene hydrate	0.32	1070
1,8-Cineole	0.34	1025
α-Pinene	0.36	972
β-Myrcene	0.39	988
3-Carene	0.46	1016
a-Selinene	0.54	1496
3-Octanone	0.58	956
3-Octanol	0.69	993
Limonene	0.74	1033
Aromadendrene	0.86	1447
Caryophyllene oxide	0.95	1587
Apofarensol	0.96	1582
Linalool	1.05	1098
4-Terpineol	1.14	1183
Thymol acetate	1.42	1354
Thymol methyl ether	1.61	1235
α-Terpinolene	1.88	1196
β-Caryophyllene	2.06	1427
*ϱ*-Cymene	10.65	1035
Carvacrol	25.96	1297
Thymol	45.42	1288
Total	98.81	

a Retention index on an HP-5 column

### Efficacy of essential oil from Z. multiflora against acute toxoplasmosis

**Fig. 1:**
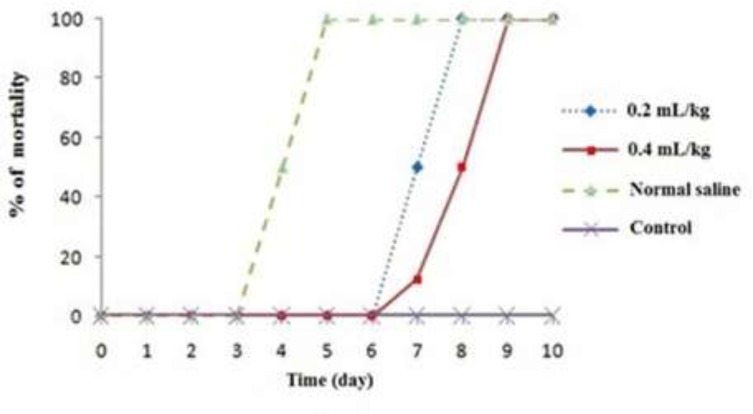
Effect of oral administration of essential oil from *Z. multiflora* on mortality rate of mice with acute toxoplasmosis infected with *T. gondii* RH strain

As shown in Fig. 1, although the mice in control group 1 did not show any mortality; but the mortality rate of the infected mice in control group 2 was 100% on the 5 ^th^ day. In contrast, 100% mortality was observed on the 8 ^th^ and 9 ^th^ d after oral administration of ZM-EO at the concentrations of 0.2 and 0.4 mL/kg, respectively. ZM-EO significantly increased the survival time in the infected mice by acute toxoplasmosis at the abovementioned doses (*P*<0.05). The average number of the tachyzoites in the infected mice cured with 0.2 and 0.4 mL/kg of ZM-EO was 189×10 ^4^ and 76×10 ^4^ parasite/mL, respectively.

In the control group, this rate was 288×10 ^4^ parasite/mL. The difference in the mean number of the tachyzoites between the infected mice that received ZM-EO at the concentrations of 0.2 and 0.4 mL/kg and the control mice was statistically significant (*P*<0.05).

### Clinical chemistry and hematological study

No mortalities were observed in the mice receiving ZM-EO at the concentrations of 0.2 (3 group) and 0.4 mL/kg (4 group) after 14 d.Tables 2 and 3 demonstrate the obtained findings of the biochemical and hematological factors after the orally treated with ZM-EO.There was no remarkable change between the treatment of mice with ZM-EO at 0.2 and 0.4 mL/kg working doses and the control group.

**Table 2:** Clinical chemistry parameters in mice sera treated with essential oil from *Z. multiflora*. (Mean ± SD).

***Parameters***	***Z. multiflora essential oil***		

	***(ml/kg)***		***Control***
	0.2	0.4	
AST (U/L)	117.6 ± 6.5	132.3 ± 13.3	124 ± 11.5
ALT (U/L)	91.2 ± 6.6	77.3 ± 5.6	80 ± 12.3
ALP (U/L)	259.3 ± 24.6	287.3 ± 22.5	280 ± 16.5
Cr (mg/dL)	0.38 ± 0.07	0.43 ± 0.08	0.4 ± 0.05
BUN (mg/dL)	59.3 ± 7.1	72.1 ± 6.6	61.4 ± 6.15
TB (mg/dL)	0.7 ± 0.23	0.66 ± 0.15	0.89 ± 0.1
DB (mg/dL)	0.08 ± 0.01	0.09 ± 0.02	0.1 ± 0.015

AST, aspartate aminotransferase; ALT, alanine aminotransferase; ALP, alkaline phosphatase; Cr, creatinine; BUN, Blood urea nitrogen; TB, Total bilirubin; DB, Direct bilirubin.

**Table 3:** Hematology parameters in whole blood of mice treated with essential oil from *Z. multiflora* (Mean ± SD).

***Parameters***	***Z. multiflora essential oil***		

	***(ml/kg)***		***Control***
	0.2	0.4	
RBC (×l0^6^/μL )	3.7 ± 0.51	4.1 ± 0.33	3.4 ± 0.3
HGB (g/dL)	9.4 ± 1.23	9.7 ± 1.07	11.3 ± 0.71
Hct (%)	25.3 ± 3.5	34.2 ± 5.1	30.3 ± 1.15
WBC (×l0^3^/μL )	2.8 ± 0.33	3.1 ± 0.43	2.5 ± 0.2
PLT (×l0^3^/μL )	307.6 ± 16.6	296.3 ± 21.3	`319.3 ± 28.2

RBC, red blood cell; HGB, hemoglobin; Hct, hematocrit; WBC, white blood cell; PLT, platele lkanes

## Discussion

Since ancient times, herbs and spices have been used for food and therapeutic purposes worldwide (9). Recently, increasing consumer concerns toward synthetic drugs have generated rising interest in a range of herbal antimicrobials agents such as essential oils (20, 21). Spices and medicinal plant essential oils and ingredients exhibit antibacterial, antifungal and antiparasitic effects against a wide spectrum of microbial pathogens (22, 23).

Nowadays, first-line chemotherapy for toxoplasmosis has a constraint in use because of some serious complications predominantly in the patients with an impaired or weakened immune system (7).

In the current study, the efficacy and safety of ZM-EO on the infected mice with acute toxoplasmosis were evaluated. The mortality rate of the infected mice in the control group was 100% on the 5th day. Surprisingly the oral administration of ZM -EO significantly increased the survival time three to four d in the infected mice by acute toxoplasmosis; so that all mice died on the 8th and 9th days after two weeks oral administration of ZM-EO at the concentrations of 0.2 and 0.4 mL/kg. Moreover, in the infected mice which received ZM-EO at the concentrations of 0.2 and 0.4 mL/kg, the mean number of tachyzoites was significantly reduced in comparison with the control group.

Recently, both innate and adaptive immune responses (humoral and cellular immunity) are necessary for control and resistance to toxoplasmosis (24). *Z. multiflora* stimulates the phagocytosis, potentiating Th _1_ , and humoral immune responses (23–26). Moreover, the immune modulatory activity of *Z. multiflora* were enhanced IFN-γ level in vitro and in vivo (27). Therefore, increased survival time in the infected mice, which received ZM-EO, may be related to the immunomodulatory properties of this plant better control measures against toxoplasmosis.

Concerning the toxicity of ZM-EO, since the liver and renal enzyme activities such as ALT, AST, ALP, bilirubin (total, direct), Cr and BUN as well as hematological parameters are the main characteristics of the liver and renal function, we evaluated the clinical and hematological parameters in the treatment of the mice receiving ZM-EO for two weeks to assess the toxicity of ZM-EO*.* Our results revealed there was no remarkable difference between the treatment of mice with ZM-EO at 0.2 and 0.4 mL/kg working doses and the control group. Therefore, referring to the toxicity classification, ZM-EO had no important toxicity against male mice (28).

Anti-*Toxoplasma* effects of a wide range of medicinal herbal derivatives such as *Vernonia colorata* Willd. (Compositae), *Zingiber officinale* Roscoe*.* (Zingiberaceae), *Sophora flavescens* Aiton. (Fabaceae), *Torilis japonica* Houtt*.* (Apiaceae), *Curcuma longa* L. (Zingiberaceae), *Juniperus procera* Hochst. Ex Endl. (Cupressaceae), and *Bunium persicum* Boiss. (Apiaceae) have been studied around the world (29, 30). However, the development of safe and efficient agents for prophylactic and therapeutic purposes for toxoplasmosis is of principal importance, which needs a wide-ranging study of medicinal plants having the capability to destroy the parasite with minimum or no toxicity to humans. The findings of this investigation recommended that ZM-EO could be a natural resource for producing a novel anti-*Toxoplasma* drug applicable in the treatment procedures of toxoplasmosis. Thus, a wide range of pharmacological properties such as antinociceptive, antioxidative, spasmolytic, anti-inflammatory, antiseptic, analgesic, antidiabetic, antifungal, and antibacterial effects have been associated with different parts of *Z. multiflora* (10, 11).

Regarding the *Z. multiflora* antiparasitic properties*,* several studies have reported the anti-protozoa and anti-helminthic activities of this plant; for example, *Z. multiflora* could eliminate the clinical symptoms in thepatients with trichomoniasis (31). The leishmanicidal activity of *Z. multiflora*; the IC _50_ values for the essential oil and methanol extract were 3.2 and 9.8 μg/mL against promastigote forms and 8.3 and 34.6 μg/mL against amastigote stages of *Leishmania tropica,* respectively (17). In the study on the in vivo efficiency of ZM-EO on hydatid cysts, ZM-EO possessed a ruinous property on the germinal layer of hydatid cyst. They also proposed that *Z. multiflora* was probably an origin of helpful constituents that might be applied in effectual antihydatid drugs (32).

A large number of investigations have considered the chemical composition of ZM-EO, commonly the richest components are oxygenated monoterpenes, monoterpene hydro-carbons and sesquiterpene hydrocarbons such as thymol and carvacrol (11, 13). By GC/MS, we found that the main compounds of ZM-EO were thymol (45.4%), carvacrol (23%) and *p*-cymene (10.6%), respectively. However, geographical difference, cultivar variations, preparation method, and further elements can manipulate the chemical composition of plants (14, 15).

The obtained results indicated that these phytocomponents in ZM -EO might account for their anti-*Toxoplasma* properties although their accurate manner of action is weakly unknown. Nevertheless, considering the antimicrobial mechanisms of terpenoid constituents such as monoterpenes, these compounds disseminate into microbes and break cell wall construction. The antimicrobial properties of terpenes are correlated with the change in permeability as well as other functions, e.g. invade into the cell and affecting some vital intracellular organelles (33, 34).

With respect to the immune-stimulatory activity, previous studies have demonstrated that ZM-EO stimulate innate immunity through increasing phagocytosis and antibodies production; therefore, the anti-*Toxoplasma* efficacy of ZM-EO through stimulating immune responses could be suggested (25, 35).

## Conclusion

The efficacy of EO-ZM at the doses 0.2 and 0.4 mL/kg with no significant toxicity to treat the acute toxoplasmosis in the mice model. Medicinal herbs could be beneficial in folk medicine to prevent and treat parasitic infections. However, additional investigations are mandatory to assess the precise effect of *Z. multiflora* essential oil, mainly its immunomodulatory, on toxoplasmosis.
